# A Longitudinal Experimental Study Examining How and Whether Practicing Acts of Kindness Affects Materialism

**DOI:** 10.3390/ijerph192316339

**Published:** 2022-12-06

**Authors:** Dariusz Drążkowski, Radosław Trepanowski

**Affiliations:** Faculty of Psychology and Cognitive Science, Adam Mickiewicz University, 89 Szamarzewskiego Street, 60-568 Poznan, Poland

**Keywords:** kindness interventions, materialism, satisfaction with life, self-determination theory

## Abstract

(1) Background: Kindness interventions assist individuals in the pursuit of greater well-being. However, little is known about whether these interventions can decrease materialism. The current study tested how kindness interventions decrease materialism and external aspirations. Furthermore, we tested whether these interventions influence impulsive shopping. (2) Method: We randomly assigned 122 females to a three-week intervention of practicing acts of kindness or a neutral intervention (practicing acts related to studying). Before and after the interventions, all participants reported their life satisfaction, level of materialism, and internal and external aspirations. (3) Results: Among women practicing acts of kindness, materialism and life satisfaction did not change compared to the control group, but in both conditions, life satisfaction increased, and materialism decreased. However, we found that practicing kindness was associated with (a) an increase in aspiration affiliation, (b) a reduction in the intention to shop impulsively, (c) less focus on external aspirations, and (d) more focus on internal aspirations. (4) Conclusions: Although our results show that practicing kindness does not lead to a decrease in materialism, they suggest that focusing on increasing personal happiness might lead to such a decrease. Furthermore, our research contributes to the existing literature by demonstrating that kind women are less oriented toward materialistic values.

## 1. Introduction

Researchers have identified many negative consequences of materialism. Materialists, compared to non-materialists, are less happy [1,2], have less satisfying social and family relationships [3], are less satisfied with their health and physical condition [4], are more anti-environmental [5], and experience financial problems more often [6]. Since materialism can be a threat to psychological health, it can be considered a public health problem [7]. According to self-determination theory (SDT), materialism negatively affects well-being because it leads to the pursuit of external aspirations (e.g., financial success). These aspirations do not directly fulfill intrinsic aspirations (e.g., social affiliation), which are crucial for well-being [8]. Therefore, practicing activities aimed at fulfilling internal needs should weaken the focus on external, materialistic aspirations. Such activities can include acts of kindness, which guide an individual’s actions to foster relationships [9] and increase life satisfaction [10]. In turn, an increase in life satisfaction can lead to a decrease in materialism [11,12]. Thus, we investigated whether practicing acts of kindness influences a decrease in materialism and external aspirations by enhancing affiliation aspirations and increasing life satisfaction. Our results may contribute to a better understanding of the relationship between kindness and materialistic attitudes and consumption behaviors. This study is of great practical importance, as a researchers should focus on developing evidence-based measures to reduce materialistic attitudes, which may yield many personal, social, and health benefits [1] and can improve public health [7].

### 1.1. Materialism and Well-Being

Richins and Dawson [13] highlighted three main dimensions of materialism: (1) material possessions as a definition of success, (2) material possessions as a source of happiness, and (3) the centrality of material possessions in one’s life. Materialism is expressed, among other ways, in a higher frequency of impulsive purchases; that is, purchases made according to a powerful urge to make them immediately without careful consideration of the consequences [14,15].

A comprehensive meta-analysis by Dittmar et al. [3] demonstrated a clear, consistent negative association between materialism and personal well-being that was stable across different operationalizations of the construct and different personal and cultural characteristics. On average, materialists, compared to non-materialists, are less satisfied with their lives, relationships, friendships, and living standards [1,2,4,13,16,17]. Materialists, compared to non-materialists, are more depressed [18], feel less meaning in life [19], and experience greater anxiety [20]. Since high materialism may impair mental health and the social functioning of individuals, which are important in the perspective of public health, it can be perceived as a public health threat [7,21,22].

One theory that explains why materialists are less happy in comparison to non-materialists is SDT [23]. This broad theoretical framework systematically explains human motivation, needs, and well-being dynamics. SDT distinguishes between two basic types of motivation: intrinsic (the inherent tendency to seek out novelty, challenges, enjoyment, and the extension and exercise of one’s capacities) and extrinsic (the performance of an activity for reasons other than inherent satisfaction). Kasser and Ryan [18,24] assumed that SDT links these two types of motivation with different aspiration types. According to them, internal aspirations are personal development, self-acceptance (a sense of competence and autonomy), relationships with others (satisfying relationships with family and friends), communality (the desire to actively and productively work for the global good), and health (the absence of illness or any health complaints). External aspirations include wealth (financial success and possession of material goods), fame or popularity (being famous, recognized, and admired), and attractiveness (fashionable clothes, an appealing face, and an attractive physique). The pursuit of external aspirations is closely linked to the possession of materialistic attitudes and values [19].

A person focused on external aspirations pursues activities related to earning rewards and being appreciated by others. In contrast, a person focused on intrinsic aspirations pursues activities that align with universal psychological needs, which provide satisfaction and lead to a greater sense of happiness than a focus on external aspirations. Past research has confirmed these assumptions by showing that people who predominantly adhere to extrinsic aspirations (relative to intrinsic aspirations) have a lower level of well-being [8,17,19,25]. Individuals who acquire material possessions to become happy are less concentrated on performing activities to achieve internal aspirations, and as a result, they experience a lower level of well-being [18,24]. Longitudinal studies have shown that people’s well-being deteriorates as they place relatively more importance on materialistic, external aspirations and values [1]. Based on this reasoning, it can be expected that encouraging individuals to be more intrinsic aspiration–oriented will increase their happiness and make them less external aspiration–oriented, which is associated with a reduction in materialism. 

### 1.2. Kindness, Materialism, and Life Satisfaction

Kindness refers to actions intended to benefit others [26,27]. No other study has directly examined the relationship between kindness and materialism. However, the relationships between materialism and constructs opposite to kindness, such as selfishness, are well documented [13,17]. For instance, previous research has found that materialists are less likely to be charitable and generous and to perform volunteer work [13,16,17]. In general, materialism harms relationships [17] and reduces cooperation [28].

Furthermore, materialism causes the erosion of friendships and prosocial behaviors by fostering the viewpoint that people, like items, exist for the benefit of others. Perhaps concentrating on material goods makes high materialists less open to the needs and problems of other people. Thus, it could be expected that kindness and materialism are negatively related. The above analysis indicates that there may be direct links between kindness and materialism, but mediating mechanisms can also be expected. 

First, both kindness and materialism show relationships to life satisfaction. A recent systematic review and meta-analysis demonstrated that performing acts of kindness significantly improves well-being [10]. Performing daily acts of kindness can increase life satisfaction [29]. Different types of kindness can lead to increased well-being, e.g., being kind to others, being kind to yourself or actively observing the kindness happening around us [30]. Acts of kindness are likely to contribute to well-being when they are varied (not repeated) [31] and when they are autonomous (not forced) [32,33]. Performing acts of kindness can lead to an increase in well-being by satisfying the fundamental psychological needs from the SDT: autonomy, competence, and relatedness [34]. Evolutionary psychology, in addition, explains the relationship between kindness and life satisfaction [10] by positing that actions focused on survival and reproduction will produce an intrinsic reward in the form of increased happiness [30]. Several evolutionary theories explain why being kind, as expressed in helping others, can increase the chances of survival and reproduction [35]. Hence, evolution ”rewards” kind individuals with an increase in happiness, to increase their chances of survival and reproduction. Thus, kindness may be at least partially genetically determined and innate [10]. These evolutionary roots of kindness have become the basis for diverse cultural norms that promote kindness between different peoples [36]. Therefore, kindness also depends on the culture and the historical time in which the individual lives. Regardless of the evolutionary and cultural determinants of kindness, research results on the intentional and conscious practice of acts of kindness shows that individuals can influence their own development of kindness [10].

Practicing acts of kindness, as described above, leads to an increase in life satisfaction. In addition, as other studies have shown, an increase in life satisfaction, e.g., through practicing gratitude (which arises when individuals receive acts of kindness from others) [26], can decrease materialism [11,12]. Thus, practicing kindness can increase life satisfaction, leading to a decrease in materialism. This leads to the following hypothesis:

**H1:**  *Performing acts of kindness (vs. control activity) increases life satisfaction (**H1a**) and decreases materialism (**H1b**), external aspirations (**H1c**), and the intention to buy impulsively (**H1d**).*

**H2:**  *Performing acts of kindness (vs. control activity) increases life satisfaction, which in turn decreases materialism (**H2a**), external aspirations (**H2b**), and the intention to buy impulsively (**H2c**).*

Second, SDT offers an alternative explanation for the effect of kindness on materialism. According to SDT [8,18,24], kindness can be considered as strongly related to intrinsic aspirations, especially affiliation aspirations. As previous research has shown, kindness may protect against the degradation of close social bonds, as it relates to feeling connected with other people [37] and can foster relationships [9]. Assuming that an increase in focus on internal aspirations decreases focus on external aspirations, practicing acts of kindness through an increased focus on affiliative aspirations can be expected to contribute to a reduction in emphasis on external aspirations, which will be associated with a decline in materialism. Building on the above reasoning, the following hypothesis is offered:

**H3:**  *Performing acts of kindness (vs. control activity) increases affiliation aspirations.*

**H4:**  *Performing acts of kindness (vs. control activity) increases affiliation aspirations, which in turn decreases materialism (**H4a**), external aspirations (**H4b**), and the intention to buy impulsively (**H4c**).*

### 1.3. The Current Study

The primary objective of our research was to explore the links between kindness and materialism. More specifically, we examined whether practicing acts of kindness decreases materialism, external aspirations, and the intention to buy impulsively. We expected these relationships to be mediated by increased life satisfaction and a focus on affiliation aspirations. Thus we applied a mediation approach, which allowed us to understand the psychological processes through which the independent variable (i.e., practicing acts of kindness) affects dependent variables (i.e., materialism, external aspirations, and the intention to buy impulsively) [38]. As we have described, the theoretical basis for the relationship between practicing acts of kindness and life satisfaction, as well as life satisfaction and materialism, can predict the presence of an indirect effect. We employed a method of the practice of acts of kindness developed within the paradigm of positive interventions, whose effectiveness in enhancing life satisfaction has been confirmed in previous studies [10]. Using the positive intervention paradigm, it is possible to apply the practice of kindness to reduce materialism in everyday life. To verify our hypotheses, we designed an experimental study with two conditions: an experimental (practicing acts of kindness) and a control (practicing neutral activity) condition. We assessed levels of materialism, life satisfaction, and intrinsic and extrinsic aspirations before and after the experimental manipulation. However, we measured kindness only at the pretest to ensure that there were no differences in its level between the intervention and control conditions. In addition, we used a vignette to measure the propensity to make an impulsive, unnecessary purchase in the post-test only.

## 2. Materials and Methods

### 2.1. Participants and Procedure

We conducted a longitudinal intervention study. As the study was conducted in a social sciences faculty with a low percentage of male students, it was decided to include only females. In this way, a non-proportional sex distribution among participants was avoided at the cost of limiting the representativeness of the results to only females. In the pretest, 156 female students aged 18–39 (*M* = 20.95, *SD* = 2.70) participated. All participants had completed secondary education and were in the process of acquiring higher education credentials. Of the participants, 122 (79%) remained in the study and participated in the post-test. To recruit prospective participants, we sent study invitations to the first- and second-year female students of the Faculty of Social Science at Adam Mickiewicz University in Poznan, Poland. The data are freely available in the Open Science Framework: https://osf.io/bc67z/?view_only=bf4933a3537c4fb3be29baec43a5b761, accessed on 6 October 2020. All procedures performed in the study followed the ethical standards of the Ethical Committee of the Faculty of Psychology and Cognitive Science, Adam Mickiewicz University in Poznań. All participants provided written informed consent. Participants were informed that the study aimed to investigate the factors influencing the effectiveness of exercises in increasing happiness. The participants who finished the study received a cinema voucher as promised. The voucher was used as an incentive to participate in the study.

There were six steps to the study: (1) pretest + first intervention (counting acts of kindness), (2–5) interventions (practicing and counting acts of kindness), and (6) post-test. The pretest was carried out during lectures at the university. After the pretest and before the first intervention, participants were randomly assigned to either the kindness intervention or active-placebo control activities and were emailed a link to a dedicated website with further instructions. The instructions for each condition that the participants were presented with are available in the supplementary file.

The participants received a message every three days inviting them to participate in the next step of the study. The participants were asked to complete the intervention (perform up to five acts of kindness or perform up to five activities related to studying) on the day they received the message or the day after. As a result, we collected data from most of the participants’ activities in both conditions every 3–4 days. Reminders prompting the completion of the exercises were sent the day after the beginning of each step. On average, it took 21 days to complete the intervention. Verifications of whether interventions were implemented were made by analyzing the content of activities described in both conditions on a dedicated website. After omitting two interventions, participants were removed from the study. At the end of the study, all participants were debriefed.

To increase the effectiveness of the kindness intervention, the participants were encouraged to perform a diverse range of acts of kindness and, along with a link to each subsequent step of the study, support messages from fictional participants of previous studies were sent. This method for enhancing the intervention and the created messages were based on the research of Nelson et al. [32], where the authors developed a six-week-long intervention. Performing acts of kindness has been shown to increase happiness in participants in interventions lasting from one day [39] to 10 weeks [40]. In general, positive interventions are more effective if they last longer [41,42]. Because the recruitment process of the study participants (students) was stretched over several weeks, we were able to design a 3-week intervention so that the last recruited participant completed participation in the study before the start of the examination session. At each step, we provided the participants with a different message, for example:

Kindness condition: Hey! You can study anywhere and anytime! You’ll surely have plenty of opportunities!

Control condition: Hey! You can do acts of kindness anywhere and anytime! You’ll surely have plenty of opportunities!

### 2.2. Measures

*Material Values Scale—Short Form* [43,44]. The scale consists of 15 items that measure “the importance ascribed to the ownership and acquisition of material goods” [43] (p. 210). The items are rated from 1 (strongly disagree) to 5 (strongly agree): for example, “I admire people who own expensive homes, cars, and clothes” (current Cronbach *α* = 0.85).

*Satisfaction with Life Scale (SWLS)* [45,46]. The SWLS is a five-item scale that measures general life satisfaction. The items are rated from 1 (strongly disagree) to 7 (strongly agree) (current Cronbach α = 0.84).

*Kindness* [26]. The scale of three items refers to the motivation, recognition, and behavior components of kindness, e.g., “I am always thinking that I wish to be kind and help other people in daily life”. The items are rated from 1 (not at all) to 5 (a great deal) (Cronbach *α* = 0.73). This scale was used only in the pretest.

*Aspiration Index* [24,47]. The Aspiration Index is a 35-item scale. Each subscale (current Cronbach alphas: self-acceptance, *α* = 0.70; affiliation, *α* = 0.91; community feeling, *α* = 0.80; social recognition, *α* = 0.90; appealing appearance, *α* = 0.78; financial success, *α* = 0.90; physical fitness, subscale omitted) consisted of five items. We calculated an extrinsic aspiration index (the relative centrality of extrinsic values, with a high score reflecting increased materialism) by subtracting the importance a subject placed on all six aspirations from the importance that the individual placed on the three extrinsic domains [20]. 

*Intention to make an impulsive purchase.* A self-designed four-item scale (e.g., ”I would buy those shoes”) rated on a 7-point Likert scale, with different anchors in each question used to measure the intention to purchase an item (current Cronbach *α* = 0.92). The item in question could be exchanged for a different one if needed. The current study measured the probability of shoe purchases in an imagined situation. To describe this situation, we used the following vignette created by Peifer, Chugani, and Roos [48]: 

Imagine you are walking past a store and happen to see an attractive pair of casual shoes in the window. They cost about as much as you’d expect them to. You already own a good pair that you are happy with, but you love the style of the new ones you see.

*Demographic questionnaire.* The participants were asked about their age and education level.

## 3. Results

The results demonstrated that before the intervention, the level of kindness was comparable in the kindness condition (*M* = 3.83, *SD* = 0.60) and the control (*M* = 3.85, *SD* = 0.66), *t*(120) = −0.14, *p* > 0.05. There were also no significant differences in the pretest measurements of other study variables, all *p* ≥ 0.10. 

We conducted correlation analysis of the relationships of kindness traits measured at the pretest with the other variables measured at the post-test in order to explore the predictive value of kindness (see Table 1). For simplicity of the data presentation and due to the lack of hypotheses regarding the effect of the experimental manipulation on the relationships between kindness and the other variables, we present the combined data for both conditions in Table 1. Kindness had significant positive correlations with life satisfaction (*r* = 0.30, *p* < 0.001), internal aspirations (*r* = 0.30, *p* < 0.001), and affiliative aspirations (*r* = 0.37, *p* < 0.001) and significant negative correlations with materialism (*r* = −0.21, *p* < 0.05), external aspirations (*r* = −0.21, *p* < 0.05), and intention to shop impulsively (*r* = −0.23, *p* < 0.01). Tables presenting correlations between all study variables separately for each condition can be found in the supplementary materials (see Tables S1 and S2).

We used repeated measures ANOVA to examine hypotheses 1 and 3 for dependent variables (life satisfaction, materialism, external aspirations, affiliative aspirations) with one between-subject factor (practicing acts of kindness group vs. active placebo control group), and one within-subject factor (before and after the intervention). Furthermore, for exploratory purposes, we included components of internal (self-acceptance, community feeling) and external aspirations (social recognition, appearance, financial success) as dependent variables. The results of these analyses are presented in Table 2. We examined hypothesis 1d using the Student’s t-test due to the lack of a pretest for the intention to buy impulsively.

We found a significant main effect of time on life satisfaction, *p* < 0.001, *η_p_*^2^ = 0.27 (large effect); materialism, *p* < 0.001, *η_p_*^2^ = 0.10 (medium effect); community feeling, *p* < 0.05, *η_p_*^2^ = 0.05 (small effect); and financial success, *p* < 0.05, *η_p_*^2^ = 0.03 (small effect). Specifically, the intervention recipients in both groups showed increased life satisfaction and community feeling and decreased materialism and financial success over time. All other main effects of time were nonsignificant, *p* > 0.05.

There was a significant interaction between time and kindness intervention on (1) affiliation, *p* < 0.05, *η_p_*^2^ = 0.05 (small effect); and (2) appealing appearance, *p* = 0.05, *η_p_*^2^ = 0.03 (small effect). Participants who performed the kindness intervention had higher affiliation on the post-test than on the pretest (supporting H3). The level of appealing appearance did not significantly change over time in the experimental group. All other interactions of time and kindness intervention were nonsignificant, *p* > 0.05 (contrary to H1a,b,c). There were no significant differences in the declared level of intention of impulse shopping, *t*(120) = 0.14; *p* > 0.05, between participants in the experimental group (*M* = 3.68, *SD* = 1.46) and participants in the control group (*M* = 3.64, *SD* = 1.45) (contrary to H1d). 

To examine hypotheses 2 and 4, three mediation analyses (Model 4) were performed using the PROCESS macro v.4.1 [49]. We chose mediation analysis, as it allows testing of how a causal antecedent directly affects a variable; when the mediating variable is causally located in-between them, the indirect effects are tested [49]. The indirect effects were tested with bias-corrected bootstrapping (*n* = 5000) and 95% confidence intervals (CIs). The partially standardized indirect effects estimated the effect size of the mediated relationship. We introduced type of intervention (practicing acts of kindness group vs. active placebo control group) as the independent variable, two mediators (changes between the pretest and post-test in life satisfaction and affiliative aspirations) and one covariate (age) into each of the three models for the dependent variables (changes between the pretest and post-test in materialism and external aspirations, intention to buy impulsively). The results of the analyses showed that models exploring changes between the pretest and post-test in materialism, *F*(4, 117) = 0.94, *p* > 0.05, *R*^2^ = 0.03, and intention to buy impulsively, *F*(4, 117) = 1.15, *p* > 0.05, *R*^2^ = 0.04, were nonsignificant (contrary to H2a,c, H4a,c). The model for changes between the pretest and post-test in external aspirations was significant, *F*(4, 117) = 31.06, *p* < 0.001, *R*^2^ = 0.52. The partially standardized indirect effects of kindness intervention on changes in external aspirations via changes in affiliative aspirations (*β* = 0.031, *SE* = 0.132, 95% *CI* = [−0.226, 0.291]) and changes in life satisfaction were nonsignificant (*β* = 0.005, *SE* = 0.014, 95% *CI* = [−0.025, 0.035]) (contrary to H2b, H4b). 

## 4. Discussion

The present study examined whether and how practicing acts of kindness influences materialism. The study results showed that practicing acts of kindness does not affect materialism, external aspirations, impulsive shopping, and life satisfaction to a greater extent than practicing neutral acts. We found that the women participating in the kindness intervention reported more affiliation values than did the controls (according to H3). However, we found that the intervention recipients, after the intervention and regardless of study condition, exhibited increased life satisfaction and community feeling aspiration and decreased materialism and financial success aspiration. This result suggests that the neutral activity in the control condition—practicing studying-related activities—and practicing kindness had similar effects on the study variables, which does not allow us to reject our hypotheses (H1, H2, and H4) entirely. Finally, we found that kind women focus less on external aspirations and more on internal aspirations. Kindness also predicted a lower intention to shop impulsively three weeks later. Overall, although our study was not conclusive as to whether practicing kindness led to a decrease in materialism, it did indicate that kind women are less oriented toward external materialistic aspirations and more toward internal aspirations, especially those related to maintaining good relationships with other people.

We hypothesized that practicing kindness could increase life satisfaction and affiliative aspirations, which would lead to a decrease in materialism, external aspirations, and a willingness to make an impulsive purchase. On one hand, our study’s results did not directly support these hypotheses. On the other hand, our findings indicated that the practice of kindness is comparable with the practice of neutral acts (such as studying) in increasing life satisfaction and decreasing materialism and financial success aspirations (an example of external aspirations). Indeed, in both conditions after the intervention, a significant increase in life satisfaction (+27%; large effect size), as well as a significant decrease in materialism (−10%; medium effect size) and financial success aspirations (−5%; small effect size), when compared to the initial measurement, were observed. The changes mentioned above may be an effect of factors unrelated to the study (e.g., changes in the weather). However, their strength (expressed in effect sizes) and consistent direction suggest that some of these changes were caused by the exercises performed by the participants in both conditions. We designed activities in the kindness and control groups based on the research methodology of Nelson et al. [32], where intervention resulted in a greater increase in well-being in the kindness condition compared to the control. Thus, perhaps engaging in internally motivated activities aimed at increasing personal happiness (the aim of the study as presented to participants), regardless of whether they consist of performing acts of kindness or taking neutral actions (performing activities related to studying), caused an increase in life satisfaction and a decrease in materialism and financial success aspirations. Moreover, a recent study demonstrated that motivating individuals to initiate value-related behavior enhances their well-being [50]. If education was an important value for our study participants, then motivating them to study harder may have contributed to an increase in their happiness. Finally, the intervention we developed differed quantitatively from that used by Nelson et al. [32]. In their study, a six-week intervention was implemented, during which participants were instructed to perform five acts of kindness every seven days (30 in total). We designed a three-week intervention, during which participants had to perform five acts of kindness every three days (20 in total). It may be that the duration of our intervention was too short; thus, the total number of acts of kindness might have been insufficient, or the greater intensity of our intervention influenced the fact that we did not observe an effect of the kindness intervention on life satisfaction.

This explanation suggests that practicing acts of kindness could eventually affect life satisfaction, materialism, and some external aspirations (financial success), but we were unable to statistically demonstrate this effect because the placebo intervention was not a completely neutral activity. This line of reasoning does not allow us to reject our hypotheses entirely, but it suggests that practicing kindness is no different from any other activity aimed at increasing personal well-being. Thus, practicing acts of kindness is likely to have the properties to increase public health by increasing life satisfaction and decreasing materialism. Future studies should examine this possibility by designing other types of neutral activities in control conditions. This is important because as research extends our knowledge about the relationship between kindness and materialism, practitioners become better equipped to help people reduce their materialism and improve the satisfaction of their lives.

Although, in general, the kindness intervention did not lead to changes in external aspirations, significant changes were observed in one of the six types of aspirations examined: we found a small effect size of practicing kindness on affiliation aspirations. As expected, participants practicing kindness showed an increase in aspirations focused on affiliation, i.e., an improvement in loving and caring for others [24]. Performing almost 20 acts of kindness within three weeks increased the importance of the aspiration of having satisfactory relations with friends and family. This relationship is consistent with our hypotheses. Following the assumptions that the realization of intrinsic aspirations (which are affiliative aspirations) leads to an increase in life satisfaction [1,8,17,19] and that an increase in life satisfaction, in turn, leads to a decrease in materialism [11,12], it can still be expected that the practicing of kindness will lead to a decrease in materialism.

Our correlational analyses of kindness as a trait measured at the pretest also confirmed this line of reasoning. We found that kindness (measured at the pretest) correlated negatively with materialism and the intention to engage in impulsive shopping (measured at the post-test). These relationships expand prior findings showing that the current level of kindness may be related to intentions to engage in impulsive shopping. From another perspective, these findings show that kinder women are less inclined toward materialistic behaviors, such as impulsively buying unnecessary items. We also found that kind women focus less on external aspirations and more on internal aspirations. The directions of the relationship with these types of aspirations are in line with our SDT-based assumptions. Being kind to other people represents an intrinsic aspiration from the SDT perspective, as confirmed by our results showing positive relationships between kindness and other intrinsic aspirations. Furthermore, kind women are less oriented toward external aspirations that do not lead to happiness. Since we have shown that there is negative correlation between kindness traits and materialism and assuming that materialism can be described in terms of the strength of extrinsic aspirations relative to intrinsic aspirations [18,24], our results suggest that kind women have fewer materialistic aspirations and values.

Since kindness interventions did not affect materialism, but kindness traits were negatively related to materialistic aspirations and values, it is possible that materialism influences kindness, but not vice versa. In line with this interpretation, a greater focus on material values may lead to a decrease in kindness toward others. Conversely, being kind to other people may not lead to a reduced focus on material things. Future research using experimental or longitudinal methodology may aim to clarify the direction of the interaction between materialism and kindness.

Despite the inconclusive results, our study was the first to test whether practicing kindness leads to a decrease in materialism and to support this hypothesis. Our line of hypotheses is supported by (1) a decrease in materialism and financial success–oriented aspirations following the use of the kindness and control interventions; (2) a significant increase in affiliation aspirations following the kindness intervention; and (3) a positive association of the trait of kindness with intrinsic aspirations and a negative association of the trait of kindness with extrinsic aspirations and with the intention to make an impulsive purchase. Thus, beyond calling for further research to test the effects of practicing kindness on materialism, initial practical recommendations can be initiated. In the modern world, many people struggle with their materialistic desires, which leads not only to a decrease in their well-being [1,3] but also to a deterioration of their social relationships [13,16,17,28], and therefore poses a threat to public health [22]. Moreover, materialistic attitudes toward buying lead to the overconsumption of goods and services, which contributes to the destruction of the environment [51]. Therefore, practitioners, especially mental health therapists, need techniques to decrease materialism. Kindness interventions, although not proven effective at this point, are a promising tool for practitioners to reduce materialism. They can be another piece in the effort to reduce the materialism of individuals for the growth of personal and social well-being and even for the improvement of public health and environmental protection.

### Limitations

There are several limitations of this study. A sample of only women was used in the study. To determine whether the same effects occur in men, additional research should be conducted to examine whether practicing acts of kindness can affect materialism by improving life satisfaction. A similar concern applies to age and education: given the high homogeneity of our sample, future research should be designed to replicate this study with participants of different ages and educational backgrounds to generalize our results to more heterogeneous populations. Second, the control task in the study seemed to increase the participants’ life satisfaction; for this reason, it may not be neutral. Future studies should consider the use of a control condition in which the participants do not perform any activity (the so-called passive placebo), with changes in well-being over time simply monitored. Third, since some of the significant results of this study had small effect sizes (e.g., the effect of kindness on affiliations), caution is needed in their interpretation as well as replication in future studies. Furthermore, future studies could control for whether practicing acts of kindness contributes to an increase in kindness—in our study, we only measured kindness as a trait at pretest to check if there were differences between study conditions. Finally, our study results could be affected by specifics of culture and economic status of the country where the study was conducted (i.e., Poland).

## 5. Conclusions

Our findings shown that practicing acts of kindness decreased materialism and external aspirations aimed at financial success. However, we observed the same effect in the control condition. Although our study is not conclusive as to whether practicing kindness leads to a decrease in materialism, it does indicate that kind women are less oriented toward external materialistic aspirations and more toward internal aspirations, especially those related to maintaining good relationships with other people, and are less willing to buy impulsively. Overall, kindness interventions are a promising tool for practitioners to reduce materialism and therefore have the potential to improve public health. We believe that our pioneering research will initiate further research to examine the impact of practicing kindness on materialism and consumption behavior.

## Figures and Tables

**Table 1:** Correlations between the variables used in the study.

	1	2	3	4	5	6	7
1. Kindness (T1)	—						
2. Life satisfaction (T2)	0.299 ***	—					
3. Materialism (T2)	−0.205 *	−0.238 **	—				
4. Intention to shop impulsively (T2)	−0.234 **	0.222 *	0.339 ***	—			
5. Affiliative aspirations (T2)	0.369 ***	0.168	−0.339 ***	−0.296 ***	—		
6. External aspirations (T2)	−0.212 *	−0.233 **	−0.022	−0.063	−0.638 ***	—	
7. Internal aspirations (T2)	0.298 ***	0.268 **	−0.270 **	−0.117	0.821 ***	−0.814 ***	—

Note: T1—pretest; T2—post-test; * *p* ≤ 0.05; ** *p* < 0.01; *** *p* < 0.001; *n* = 122.

**Table 2:** Testing the kindness intervention on life satisfaction, materialism and aspirations.

Measure	Pre-Test				Post-Test				Time	Time × Group
	Kindness (N = 61)		Control (N = 61)		Kindness (N = 61)		Control (N = 61)			
	*M*	*SD*	*M*	*SD*	*M*	*SD*	*M*	*SD*	*F*(1, 120)	*F*(1, 120)
Life satisfaction	4.10	1.12	3.77	1.20	4.54	1.26	4.34	1.18	45.13 ***	0.70
Materialism	2.77	0.61	2.71	0.65	2.68	0.63	2.61	0.68	13.26 ***	0.66
Internal aspirations	6.20	0.45	6.12	0.58	6.35	0.38	6.22	0.62	2.09	0.45
Self-acceptance	6.37	0.52	6.51	0.45	6.41	0.54	6.57	0.55	1.56	0.84
Affiliation	6.50	0.59	6.54	0.74	6.66	0.51	6.50	0.76	1.66	5.69 *
Community feeling	5.76	0.82	5.47	1.12	5.90	0.70	5.65	1.09	5.70 *	0.76
External aspirations	−7.31	0.56	−7.13	0.80	−7.44	0.52	−7.26	0.82	2.64	0.05
Social recognition	3.35	1.22	3.14	1.31	3.29	1.15	3.22	1.25	0.07	0.22
Appearance	4.26	1.19	3.93	1.10	4.12	1.13	4.04	1.02	0.05	3.77 *
Financial success	4.33	1.18	4.32	1.21	4.18	1.23	4.18	1.16	4.15 *	0.01

Note: * *p* ≤ 0.05, *** *p* < 0.001.

## Data Availability

The dataset is available for free download from the Open Science Framework (https://osf.io/bc67z/?view_only=bf4933a3537c4fb3be29baec43a5b761, accessed on 6 October 2020).

## Supplementary Materials

The following supporting information can be downloaded at: https://www.mdpi.com/article/10.3390/ijerph192316339/s1.
