# Supplementary material for: A Longitudinal Experimental Study Examining How and Whether Practicing Acts of Kindness Affects Materialism
Source: Int J Environ Res Public Health. 2022 Dec 6;19(23):16339. doi: 10.3390/ijerph192316339 (PMC9736427; doi:10.3390/ijerph192316339)
Supplement: Supplementary file 1 [file ijerph-19-16339-s001.zip › ijerph-2001023-supplementary.pdf]

Supplementary File to: A longitudinal experimental study examining how and whether practicing acts of kindness affects materialism

Table S1. Correlations between the variables used in the study in the experimental group

	**1**	**2**	**3**	**4**	**5**	**6**	**7**	**8**	**9**	**10**
1. Life sat. (T1)	-									
2. Life sat. (T2)	.739***	-								
3. Material. (T1)	-.214	-.297*	-							
4. Material. (T2)	-.298*	-.394***	.894***	-						
5. Affiliation (T1)	-.224*	-.303*	.053	-.078	-					
6. Soc. rec. (T1)	-.132	-.201	.320***	.351**	.100	-				
7. Finc. suc (T1)	.110	-.025	.609***	.537***	.156	.474***	-			
8. Comm. f. (T1)	-.031	.072	.164	-.026	.473***	.214	.338**	-		
9. Appear. (T1)	.068	-.131	.583***	.606***	.170	.404***	.626***	.395***	-	
10. Self-acc. (T1)	-.052	.069	.159	.114	.445***	.302**	.395***	.450***	.304**	-
11. Ext. asp. (T1)	.177	.226	-.267*	-.222	-.704***	-.669***	-.494***	-.718***	-.462***	-.697***
12. Int. asp (T1)	-.146	-.127	.143	-.015	.857***	.231*	.341**	.802***	.345**	.718***
13. Kindness (T1)	.055	.246	-.129	-.286*	.269*	-.030	.013	.239*	-.064	.171
14. Affiliation (T2)	-.168	-.042	-.377**	-.343**	.509**	-.228	-.227	.057	-.278*	.162
15. Soc. rec. (T2)	-.063	-.077	.370**	.422**	-.214	.717***	.527***	.092	.445***	.162
16. Finc. suc (T2)	.002	.002	.608***	.672***	-.169	.351**	.780***	.072	.577***	.255*
17. Comm. f. (T2)	.330**	.248	-.037	-.061	.091	.006	.152	.829***	.288*	.140
18. Appear. (T2)	.021	.029	.513***	.555***	-.195	.394**	.585***	.201	.800***	.262*
19. Self-acc. (T2)	.196	.285*	-.075	-.072	.063	.059	.175	.078	-.037	.637**
20. Ext. asp. (T2)	-.051	-.098	-.060	-.104	-.114	-.417**	-.369**	-.366**	-.265*	-.427**
21. Int. asp (T2)	.139	.221	-.283*	-.272*	.382**	-.109	.013	.446***	-.057	.464***
22. Int. to buy (T2)	.252*	.292*	.334**	.260*	-.427**	.176	.340**	.044	.282*	-.075

Note. T1 - pre-test (n = 78); T2 - post-test (n = 61); *$p \leq .05$; **$p < .01$; ***$p < .001$

Table S1. Cont.

	**11**	**12**	**13**	**14**	**15**	**16**	**17**	**18**	**19**	**20**	**21**
1. Life sat. (T1)											
2. Life sat. (T2)											
3. Material. (T1)											
4. Material. (T2)											
5. Affiliation (T1)											
6. Soc. rec. (T1)											
7. Finc. suc (T1)											
8. Comm. f. (T1)											
9. Appear. (T1)											
10. Self-acc. (T1)											
11. Ext. asp. (T1)	-										
12. Int. asp (T1)	-.878***	-									
13. Kindness (T1)	-.208	.292**	-								
14. Affiliation (T2)	-.139	.399**	.462***	-							
15. Soc. rec. (T2)	-.455***	-.028	-.093	-.100	-						
16. Finc. suc (T2)	-.252	.026	-.251	-.172	.531***	-					
17. Comm. f. (T2)	-.349**	.475***	.085	-.020	.142	.050	-				
18. Appear. (T2)	-.312*	.070	-.113	-.129	.600***	.707***	.220	-			
19. Self-acc. (T2)	-.271*	.319*	.000	.233	.149	.107	.175	.140	-		
20. Ext. asp. (T2)	.564**	-.395**	-.155	-.416**	-.735***	-.342**	-.452***	-.470***	-.575***	-	
21. Int. asp (T2)	-.370**	.609***	.321*	.712***	.073	-.032	.522***	.087	.695***	-.731***	-
22. Int. to buy (T2)	.080	-.271*	-.123	-.312*	.321*	.369**	.182	.274*	-.064	-.125	-.139

Note. T1 - pre-test (n = 78); T2 - post-test (n = 61); *$p \leq .05$; **$p < .01$; ***$p < .001$

Table S2. Correlations between the variables used in the study in the control group

	1	2	3	4	5	6	7	8	9	10
1. Life sat. (T1)	-									
2. Life sat. (T2)	.778***	-								
3. Material. (T1)	-.130	-.051	-							
4. Material. (T2)	-.215	-.095	.911***	-						
5. Affiliation (T1)	.084	.149	-.295**	-.303*	-					
6. Soc. rec. (T1)	.080	.141	.300**	.251	.101	-				
7. Finc. suc (T1)	-.078	.107	.699***	.656***	-.133	.350**	-			
8. Comm. f. (T1)	.003	.152	.052	-.015	.179	.030	.102	-		
9. Appear. (T1)	.033	.178	.526***	.488***	-.073	.541***	.461***	.118	-	
10. Self-acc. (T1)	-.025	.068	-.029	-.166	.291*	.063	.119	.161	.077	-
11. Ext. asp. (T1)	-.081	-.213	-.051	.042	-.658***	-.651***	-.209	-.534***	-.344**	-.466***
12. Int. asp (T1)	.045	.186	-.160	-.255*	.786***	.096	.009	.678***	.041	.564***
13. Kindness (T1)	.126	.357**	-.113	-.136	.465***	.086	.079	.309**	.065	.315**
14. Affiliation (T2)	.239	.305*	-.391**	-.365**	.754***	-.058	-.210	.259*	-.106	.228
15. Soc. rec. (T2)	.020	.182	.370**	.345**	.114	.760***	.481***	.026	.515***	.069
16. Finc. suc (T2)	-.089	.081	.711***	.706**	-.153	.336**	.822***	-.001	.463***	.042
17. Comm. f. (T2)	.087	.189	-.011	-.039	.232	.225	-.048	.717***	.191	.155
18. Appear. (T2)	.064	.294*	.467***	.463**	.039	.504***	.434***	.206	.785***	.150
19. Self-acc. (T2)	.073	.152	-.108	-.178	.347**	.034	-.032	.135	.082	.618**
20. Ext. asp. (T2)	-.167	-.335**	.015	.041	-.566**	-.456***	-.134	-.397**	-.304*	-.338**
21. Int. asp (T2)	.201	.306*	-.270*	-.288*	.651***	.069	-.155	.492***	.041	.387**
22. Int. to buy (T2)	.074	.145	.425**	.414**	-.389**	.141	.198	.033	.358**	-.013

Note. T1 - pre-test (n = 76); T2 - post-test (n = 61); $^{*}p \leq .05$; $^{**}p < .01$; $^{***}p < .001$

Table S2. Cont.

	11	12	13	14	15	16	17	18	19	20	21
1. Life sat. (T1)											
2. Life sat. (T2)											
3. Material. (T1)											
4. Material. (T2)											
5. Affiliation (T1)											
6. Soc. rec. (T1)											
7. Finc. suc (T1)											
8. Comm. f. (T1)											
9. Appear. (T1)											
10. Self-acc. (T1)											
11. Ext. asp. (T1)	-										
12. Int. asp (T1)	-.818***	-									
13. Kindness (T1)	-.460***	.538***	-								
14. Affiliation (T2)	-.458***	.685***	.332**	-							
15. Soc. rec. (T2)	-.504***	.107	.046	.117	-						
16. Finc. suc (T2)	-.123	-.092	.014	-.107	.473***	-					
17. Comm. f. (T2)	-.499***	.519***	.153	.345**	.154	-.026	-				
18. Appear. (T2)	-.397**	.159	.204	.172	.612***	.558***	.328**	-			
19. Self-acc. (T2)	-.350**	.461***	.115	.410**	.016	.004	.253*	.222	-		
20. Ext. asp. (T2)	.722***	-.650***	-.255*	-.734***	-.637***	-.194	-.623***	-.562***	-.516***	-	
21. Int. asp (T2)	-.585***	.762***	.296*	.863***	.139	-.073	.696***	.305*	.651***	-.851***	-
22. Int. to buy (T2)	.100	-.250	-.336**	-.306*	.198	.218	.189	.317*	-.021	-.019	-.110

Note. T1 - pre-test (n = 76); T2 - post-test (n = 61); $*p \leq .05$; $**p < .01$; $***p < .001$

Supplementary File 1 Experimental group instruction

ENGLISH

In recent years, psychologists have found that there are some simple exercises that can increase the levels of well-being and happiness. One of them is the "Kindness Acts List"

In our daily lives, we all perform acts of kindness (goodness) to others. These acts can be either large or small. The person for whom we perform an act of kindness may or may not be aware of the act. For instance, an act of kindness might be helping one's parents to prepare the dinner, giving way to an elderly person on the tram, washing your roommate's dishes, helping a friend study, or visiting an elderly relative.

Perform 5 good acts in the next 3 days. Please keep in mind that:

1. the acts do not have to be done for the same person

2. the person for whom you perform an act of kindness does not have to be aware of it

3. the act of kindness itself does not have to be similar to the acts listed above.

4. please do not perform any acts that may put you or others in danger.

In 3 days you will be asked to report what acts of kindness you have performed through an entry on the survey page.

Supplementary File 2 Control group instruction

ENGLISH

In recent years, psychologists have found that there are some simple exercises that can increase the levels of well-being and happiness. One of them is the "Academic Activities List"

In our daily lives, we all have certain activities that we should do, such as studying. For students, focusing on doing study-related activities on a regular basis increases well-being. This exercise involves performing study-related activities on a regular basis, such as studying, taking notes, reading literature for class or going to lectures.

Perform 5 good acts in the next 3 days. Please keep in mind that:

1. the acts do not have to be related to one subject

2. this activity can last for any length of time, i.e. either a few minutes or a few hours

3. this activity does not have to be similar to the ones listed above.

In 3 days you will be asked to report what acts of kindness you have performed through an entry on the survey page.
